# Effects of service-wide support on regularity of alcohol screening of clients in Australian Aboriginal and Torres Strait Islander Community Controlled Health Services: a cluster randomised trial

**DOI:** 10.1186/s13722-022-00294-6

**Published:** 2022-02-19

**Authors:** Monika Dzidowska, Jacques E. Raubenheimer, Timothy A. Dobbins, K. S. Kylie Lee, Noel Hayman, Julia Vnuk, Paul Haber, Katherine M. Conigrave

**Affiliations:** 1 Faculty of Medicine and Health, Discipline of Addiction Medicine, NHMRC Centre of Research Excellence in Indigenous Health and Alcohol, The University of Sydney, Lev 6, King George V Building (C39), Sydney, NSW 2006 Australia; 2 The Edith Collins Centre (Translational Research in Alcohol Drugs and Toxicology), Sydney Local Health District, Drug Health Services, Royal Prince Alfred Hospital (KGV), 83-117 Missenden Road, Camperdown, Sydney, NSW 2050 Australia; 3 Faculty of Medicine and Health, Translational Australian Clinical Toxicology Program, The University of Sydney, Lev3, 1-3 Ross Street (K06), Sydney, NSW 2006 Australia; 4 School of Public Health and Community Medicine, University of New South Wales-UNSW, Level 3, Samuels Building Gate 11, Botany Street, Sydney, NSW 2052 Australia; 5 Faculty of Health Sciences, National Drug Research Institute, Curtin University, 7 Parker Place, Bentley, Perth, WA 6102 Australia; 6 Centre for Alcohol Policy Research, La Trobe University, NR1, Bundoora, Melbourne, VIC 3086 Australia; 7 Southern Queensland Centre of Excellence in Aboriginal and Torres Strait Islander Primary Health Care (Inala Indigenous Health Service), 37 Wirraway Parade, Inala, Brisbane, QLD 4077 Australia; 8 Griffith Health Centre (G40), School of Medicine, Griffith University, Gold Coast campus, Gold Coast, QLD 4222 Australia; 9 School of Medicine, University of Queensland, Herston Road, Herston, Brisbane, QLD 4006 Australia; 10 Aboriginal Health Council of South Australia, 220 Franklin Street, Adelaide, SA 5000 Australia; 11 Adelaide Rural Clinical School, The University of Adelaide, Level 1, Helen Mayo North Frome Road, Adelaide, SA 5005 Australia

**Keywords:** Alcohol, Training and support, Alcohol screening, Screening patterns, Indigenous, Aboriginal, Torres Strait Islander, Continuous quality improvement, AUDIT-C, Primary care

## Abstract

**Background:**

We have previously shown that service-wide support can increase the odds of alcohol screening in any 2-month period in a cluster randomized trial of service-wide support to Aboriginal and Torres Strait Islander Community Controlled Health Services (ACCHS). Here we report an exploratory analysis on whether the resulting pattern of screening was appropriate. Aim: we assess whether that increase in screening was associated with: (i) increased first-time screening, (ii) increased annual screening, (iii) whether frequently screened clients fell into one of four risk categories as defined by national guidelines.

**Methods:**

Setting and participants: 22 ACCHS; randomized to receive the support model in the treatment (‘early-support’) arm over 24-months or to the waitlist control arm. Intervention: eight-component support, including training, sharing of experience, audit-and-feedback and resource support. Analysis: records of clients with visits before and after start of implementation were included. Multilevel logistic modelling was used to compare (i) the odds of previously unscreened clients receiving an AUDIT-C screen, (ii) odds of clients being screened with AUDIT-C at least once annually. We describe the characteristics of a sub-cohort of clients who received four or more screens annually, including if they were in a high-risk category.

**Results:**

Of the original trial sample, 43,054 met inclusion criteria, accounting for 81.7% of the screening events in the overall trial. The support did not significantly increase the odds of first-time screening (OR  = 1.33, 95% CI 0.81–2.18, p  = 0.25) or of annual screening (OR  = 0.99, 95% CI 0.42–2.37, p = 0.98). Screening more than once annually occurred in 6240 clients. Of the 841 clients with four or more screens annually, over 50% did not fall into a high-risk category. Females were overrepresented. More males than females fell into high-risk categories.

**Conclusion:**

The significant increase in odds of screening observed in the main trial did not translate to significant improvement in first-time or annual screening following implementation of support. This appeared to be due to some clients being screened more frequently than annually, while more than half remained unscreened. Further strategies to improve alcohol screening should focus on appropriate screening regularity as well as overall rates, to ensure clinically useful information about alcohol consumption.

*Trial Registration* ACTRN12618001892202, retrospectively registered 16 November 2018 https://anzctr.org.au/Trial/Registration/TrialReview.aspx?ACTRN=12618001892202.

**Supplementary Information:**

The online version contains supplementary material available at 10.1186/s13722-022-00294-6.

## Background

In Australia, Aboriginal Community Controlled Health Services (ACCHS) provide culturally appropriate, holistic health care and make key contributions to improving health outcomes for Australia’s Aboriginal and Torres Strait Islander peoples (also respectfully referred to as First Nations Australians) [1]. These services play an important role in addressing health inequalities experienced by their clients. The inequalities include significant harms from alcohol [2, 3]. Alcohol contributes to 8.1% of the health gap between First Nations Australians and other Australians [4]. This is despite the fact that more Aboriginal and Torres Strait Islander peoples than non-Indigenous are current non-drinkers [5], and prevalence of dependence (2.2%) is similar to the general population [6]. However, when First Nations Australians people do drink, they consume a median of 78 g of alcohol per occasion, well over the Australian recommended limit to reduce risk of short-term harms, like injury, from alcohol (40 g per occasion) [7]. These patterns of drinking and harms have roots in ongoing trauma from colonisation [8]. Other Indigenous peoples who have been colonised have suffered increases in harms from alcohol, however, there are very few studies addressing alcohol screening in Indigenous populations [9].

Australian guidelines [10] recommend that Aboriginal and Torres Strait Islander peoples are screened with a validated tool, such as the Alcohol Use Disorders Identification Test—Consumption (AUDIT-C) [11], ‘as part of an annual health assessment, or opportunistically’. As part of this same guideline, more frequent screening is recommended for high-risk groups including adolescents and young adults, those with high-risk and harmful drinking levels, those with conditions exacerbated by alcohol, and women who are pregnant or planning pregnancy [10].

Implementation studies in general primary care aim to increase the absolute number of alcohol screening events or patients screened. A recent systematic review showed that 34 of 44 studies aiming to improve alcohol screening resulted in significant increases in rates [9]. However, none investigated whether such interventions resulted in appropriate screening frequency or regularity. These studies therefore do not provide information on whether screening was clinically appropriate. For example, recommendations for universal opportunistic screening could in theory lead to unnecessarily frequent screening of clients who attend clinics often. Conversely, people with health conditions exacerbated by alcohol or people who are dependent on alcohol may need more frequent than annual screening.

In this paper we report on a secondary analysis of a cluster randomized trial of a multi-component, service-wide support for ACCHS to increase universal alcohol screening and appropriate treatment. We have previously shown that the support model could increase the odds of a person being screened with AUDIT-C when attending the participating ACCHS in any 2-month period over 24 months of implementation. The increase in odds from baseline to 24 months post-implementation was nearly eight times greater in the treatment arm (OR = 7.95, 95% CI 4.04–15.63, p < 0.001) than in waitlist controls [12]. However, that study did not investigate whether this increase resulted in clinically appropriate screening frequency or regularity.

Here we explore whether this increase resulted in patterns of screening that are in keeping with recommended guidelines. We thus examined data to answer the following questions:

1. Were previously unscreened clients more likely to be screened with AUDIT-C after support commenced?
2. Were clients in the treatment arm more likely to undergo regular annual screening?
3. What proportion of clients was frequently screened within any 12-month period and did these clients fall into high-risk categories?

## Methods

### Ethical approval and consent

This study received approval from eight ethics committees in Australian states and territories where the participating services were located: The Aboriginal Health and Medical Research Council of NSW Ethics Committee (1217/16), Central Australian Human Research Ethics Committee (CA-17-2842), Human Research Ethics Committee of the Northern Territory Department of Health and Menzies School of Health Research (2017-2737), Central Queensland Hospital and Health Service Human Research Ethics Committee (17/QCQ/9), Far North Queensland Human Research Ethics Committee (17/QCH/45-1143), The Aboriginal Health Research Ethics Committee, South Australia (04-16-694), St Vincent’s Hospital Melbourne Human Research Ethics Committee (LRR 036/17) and Western Australian Aboriginal Health Ethics Committee (Project 779).

### Study design and recruitment

The full study protocol (Trial Registration: ACTRN12618001892202, retrospectively registered) has been published elsewhere [13]. Briefly, the study is a cluster randomised trial of 22 ACCHS located across Australia equally allocated to the treatment and waitlist control arms.

### Implementation strategy

The multifaceted model of support for implementing screening and a full range of clinical responses for unhealthy alcohol use consisted of eight core components (Table 1). Screening was addressed in multiple components. Training and support emphasised annual screening of all clients aged 16 or older and discussed opportunities for screening (e.g., antenatal checks or when seeing a nurse or Aboriginal health professional), and when not to screen (e.g., in crisis situations).

**Table 1:** Description of the support model, with detail on elements relating to screening^a^

Component	Description
1	A memorandum of understanding outlining the aims of and design of the study, responsibilities of the research team and the service
2	Two-day workshop with two nominated service champions to introduce aims and methods of the study, the support model, and to build a champions’ network. Training included screening, brief intervention, and treatment of unhealthy alcohol use
3	On-site training: the core program was half-day, face-to-face workshop. Training included: harms related to alcohol; current evidence for screening; culturally secure and accurate administration and interpretation of AUDIT-C; use of annual AUDIT-C screening; responding to a positive AUDIT-C screen; and using service-wide screening data to monitor improvements in screening Implementation approaches incorporated cultural protocols of Aboriginal and Torres Strait peoples such as gender appropriateness, kinship systems and cultural obligations Face-to-face workshops were delivered by an addiction medicine specialist and an Aboriginal health professional (e.g., drug and alcohol worker or other)
4	Data feedback report, based on the bi-monthly data provided by services Graphic representation of proportion of clients screened; proportion drinking at risky levels as measured by AUDIT-C; as well as overall rate of screening over 2-month periods and the last 12-months; and recorded treatment provided Presented as a pdf file with graphics and emailed to service champions and key contacts
5	Bi-monthly teleconference for service champions to exchange improvement ideas and experiences
6	Support to modify practice software to facilitate screening such as inclusion of AUDIT-C in the Adult Health Check, and other electronic forms used for periodic and opportunistic health checks, e.g., over 50 s, pregnancy, pre-consult examination
7	A website with a repository of electronic tools and resources, including screening resources and standard drinks charts, and a private chat platform for champions
8	Financial support for purchase of agreed resources e.g., standard drink cups, clinical handbooks, prevention materials

^a^This table emphasises the screening-specific content of the support model. Fuller description, including elements supporting alcohol treatment, has been published elsewhere [12, 15]

The treatment arm received support first (the ‘early-support’ arm). The total duration of implementation in the early-support arm was 24 months, consisting of 12 months of active support (Table 1, components 1–8), followed by maintenance support of 12 months (Table 1, components 4–8). During that time the waitlist control services operated as normal and interaction with the researchers occurred only for collection of routinely collected data. At the end of that period, which marked the end of the randomised trial, the waitlist control arm began receiving the full support model [14]. Data from the waitlist control implementation phase is not reported here.

### Collaboration with Aboriginal community Controlled Health Service

ACCHS are primary health care services managed and operated by local Aboriginal and/or Torres Strait Islander communities. The study was developed and conducted in partnership with ACCHS to build on strengths and uniqueness of each service and to enhance how alcohol care is delivered locally.

### Data collection

ACCHS provided routinely collected clinical data from their electronic medical record system, Communicare, without personal identifiers, every 2 months. Records of patients who were 15 years or older were eligible for extraction. A client observation was recorded if the client had attended in the 2 months preceding extraction. Each observation included the date of the last visit in the preceding 2 months, basic demographic and health information including AUDIT-C score and date of AUDIT-C screen; systolic blood pressure (BP); haemoglobin A1c (HbA1c); and the liver enzyme, gamma-glutamyl transferase (GGT). Baseline data for 18 months prior to implementation in the early-support arm (28 February 2016–30 August 2017) was obtained for all 22 services. Records for individual clients were matched using patient IDs (with no personal identifiers attached) [13].

### Analysis

For this secondary analysis, records of patients were eligible if they had at least one visit before and one visit on or after the date when the support model started implementation (here described as ‘current clients’). This was to capture individuals who attended the service with some regularity during the trial.

#### Question 1: Screening of previously unscreened clients after implementation

**Fig. 1:**
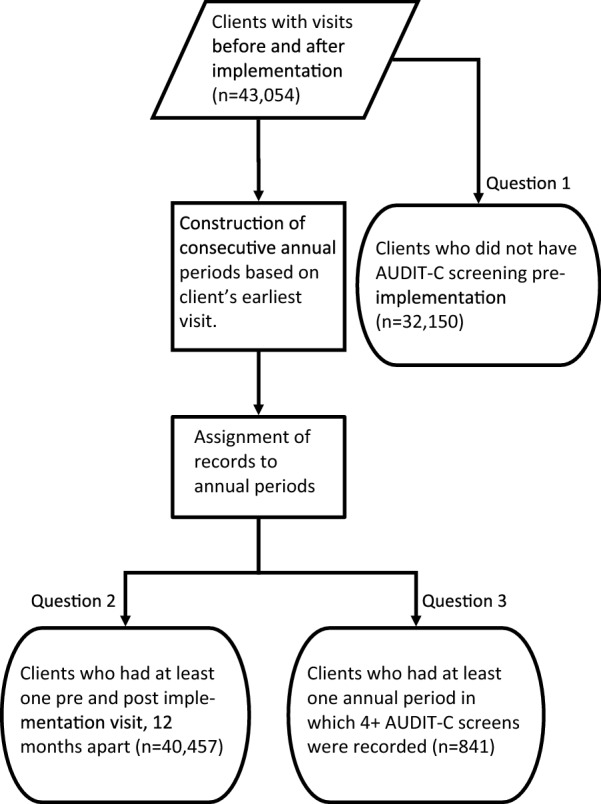
Construction of analytical samples

To address question 1, we tested whether the support model improved the odds of previously unscreened clients being screened for the first time (in the early support arm when compared to waitlist control), in the 24 months after start of implementation of the support model. For this sub-analysis, only records of patients who had not been screened in the baseline period were included (Fig. 1). The outcome measure was whether the patient had at least one recorded AUDIT-C screen on or after the start date of implementation.

#### Question 2: Systematic annual screening of clients after implementation

To address question 2, we tested if the support model improved the odds of receiving annual AUDIT-C screening post-implementation (for clients in the early versus waitlist control arms). The 18 months of available baseline data did not enable us to establish if clients were screened annually pre-implementation. However, as data from this trial showed very low rates of baseline screening [12, 15], and as AUDIT-C screening was only made a national key performance indicator for these services in mid-2017 [16], we assumed that in most services no annual AUDIT-C screening had occurred prior to implementation. Clients were eligible for inclusion in this analysis if their earliest and latest visits were at least 12 months apart. For each eligible client, consecutive 12-month intervals were constructed from the date of their earliest visit during the study. Each period was defined as occurring pre- or post-implementation based on the latest date of client visit within that period (Fig. 1 and Additional file 1: Figure S1). A 12-month period for a client with at least one AUDIT-C screen was defined as a ‘screened’ period. All other 12-month periods with at least one visit were considered ‘non-screened’ periods. Clients were considered ‘annually screened’ if all their annual periods that occurred post-implementation were screened periods.

#### Question 3: Frequent screening among low and high-risk clients

To address question 3, we investigated characteristics of clients who were frequently screened. We defined a client to be frequently screened if they had four or more AUDIT-C screens recorded in any 12-month period (Fig. 1 and Additional file 1: Figure S2). Clients were included if there was at least one annual period with visits, regardless of their timing relative to implementation. Clients’ annual periods were classified as pre-, post-, or spanning implementation based on their period start and end dates.

We investigated what proportion of these clients fell into a high-risk health category based on the clients’ earliest record of AUDIT-C, systolic blood pressure (BP), haemoglobin A1c (HbA1c) and gamma-glutamyltransferase (GGT). Clients were considered ‘high-risk’ if at least one of these results was above recommended levels:

- AUDIT-C: for Aboriginal and Torres Strait Islander populations a score of 4 or higher for males and 3 or higher for females is used to suggest risky drinking [17].
- BP: systolic BP of 140 mmHg or higher is indicative of hypertension [18].
- HbA1c: levels of 6.5% or above are indicative of diabetes [19].
- GGT: 51 U/L or above for males, and 36 U/L or above for females, is considered an abnormal liver enzyme result [20].

Analyses were conducted on an intention-to-treat basis. For questions 1 and 2 outcomes for clients within the same service were likely to be correlated. Therefore, the effect of clustering was accounted for in the analysis. We conducted multilevel logistic regression using the ‘lme4’ package [21] in the R statistical software environment, version 4.0.2 [22].

Our model incorporated the fixed effect of ‘condition’ [whether a service was assigned to the early-support (condition = 1), or waitlist control arm (condition = 0)], the random intercept of service, and controlled for age and gender.

We calculated confidence intervals for the fixed effects using Wald estimation. We estimated the effect of implementation on the early-support arm (simple slope) using the delta method (‘car’ package) [23, 24]. Adjusted Intraclass Correlation Coefficients (ICC) were calculated using the ‘performance’ package [25, 26] to describe the proportion of variability explained by differences between clusters.

Preliminary analysis showed that only 10 of the 22 services were represented in the sample of clients who received four or more AUDIT-C screens in an annual period. Statistical significance testing was therefore not conducted. Accordingly, we used descriptive statistics to explore question 3.

#### Missing data

Clients were excluded from analysis if their gender or age were not recorded. Since the study used routinely collected practice data, it was not possible to determine whether any AUDIT-C screening data were missing.

## Results

### Description of sample

Twenty-two ACCHS were recruited to the study and randomised to either early or waitlist control arms. From January 2019 onwards, one service in the waitlist control arm was unable to provide data due to a change in practice software. For the present analysis, the trial sample was comprised of 89,788 individual clients with observations between 28 February 2016 and 30 August 2019. Of these, there were 43,054 current clients (attended at least once before and once after implementation). The 46,734 clients who were excluded from these analyses accounted for 18.3% (11,163) of all AUDIT-C screening instances recorded between 28 February 2016 and 30 August 2019 (61,075). The mean age and gender distribution in study arms remained the same as in the trial (see Table 2 and Additional file 1: Table S1), indicating that sample construction processes did not disrupt the gender balance in the trial arms established by randomisation.

**Table 2:** Unscreened sample at baseline^a^: characteristics by trial arm

Characteristic	Early support	Waitlist controls
Services		
n	11	11
Mean clients per service (SD)	1986 (1109)	936 (574)
Remoteness		
Urban and inner regional	5	5
Outer regional and remote	2	3
Very remote	4	3
Clients		
n	21,850	10,300
Mean age of clients in years (SD)	36.8 (15.9)	37.5 (16.2)
Number of female clients (%)	12,412 (56.8)	5859 (56.9)
Mean observations^b^ per client (SD)	3.9 (2.6)	3.9 (2.6)

^a^Baseline period: from 28.02.2016 to 30.08.2017 inclusive

^b^An observation appeared in the dataset for a client if they attended their service for a consultation in the preceding 2-month reference period

#### Were more clients screened for the first time after implementation in the early-support arm?

Of the current clients, 32,150 had no AUDIT-C recorded pre-implementation. The baseline characteristics of this sub-sample are shown in Table 2. Two clients had missing gender and were excluded from analysis.

During the 24 months of support, 20,141 clients were not screened at all. Two in five (n = 8761 40.1%) individuals in early-support arm were screened for the first time as were 3248 (31.5%) in waitlist control arm. Controlling for age and gender did not have any significant impact on the fixed effect of condition, so the results of the simpler model are presented. The odds of a client being screened for the first time in the waitlist control arm were 0.52 (95% CI 0.37–0.74, p < 0.001). Clients in the early-support had 33% greater odds than the waitlist controls of receiving a screen for the first time but this result was not significant (OR = 1.33, 95% CI 0.81–2.18, p = 0.25). There was modest variability in the effect, with service difference accounting for 10% of the variability in the odds of being screened for the first time (Additional file 1: Table S2).

#### Were more clients screened annually after implementation?

Of the current clients, 40,457 had at least one visit to their service in a post-implementation annual period. One client had missing gender and was excluded from the analysis. The baseline characteristics of this sample are shown in Table 3. Over the 24 months of support, 3091 (11.4%) in early-support arm and 1371 (9.9%) in waitlist controls were screened annually. Although the effects of age and gender were significant, they did not alter the effect of condition. Therefore, results from the simpler model are presented. The odds of annual screening for the waitlist-control arm were 0.08 (95% CI 0.04–0.15, p < 0.001). Clients in the early-support arm had the same odds as the waitlist controls of being annually screened (OR = 0.99, 95% CI 0.42–2.37, p = 0.98) over the support implementation period. Differences between services accounted for 24% of the variance in the odds of annual screening (Additional file 1: Table S3).

**Table 3:** Annually screened sample at baseline ^a^: characteristics by trial arm

Characteristic	Early support	Waitlist control
Services		
n	11	11
Mean clients per service (SD)	2420 (1699)	1259 (521)
Remoteness		
Urban and inner regional	5	5
Outer regional and remote	2	3
Very remote	4	3
Clients		
n	26,614	13,843
Mean age of clients in years (SD)	37.2 (15.8)	37.6 (16.2)
Number of female clients (%)	15,261 (57.3)	7976 (57.6)
Mean observations^b^ per client (SD)	4.6 (2.9)	4.7 (3.1)
Clients screened with AUDIT-C (%)	6382 (24)	4009 (29)
Mean AUDIT-C score^c^ (SD)	3.9 (3.8)	3.3 (3.6)
Clients with an AUDIT-C score > 0^c^ (%)	3665 (57.4)	2482 (61.9)

^a^Baseline period: from 28.02.2016 to 30.08.2017 inclusive

^b^An observation appeared in the dataset for a client if they attended their service for a consultation in the preceding 2-month reference period at least once

^c^The denominator is the number of clients who had at least one recorded AUDIT-C score

#### What proportion of clients were frequently screened in any 12-month period?

**Fig. 2:**
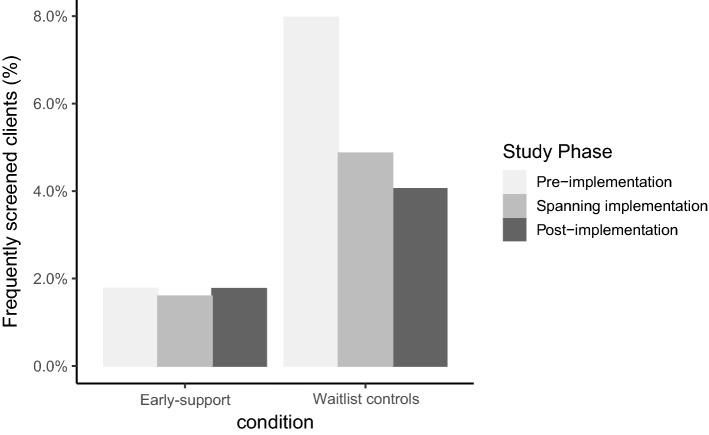
Proportion of patients with frequently screened annual periods in each study phase (n_services_ = 10)^a^. ^a^Figure shows data from 10 services where frequent screening occurred (at least one client had 4 + screens annually). Frequently screened annual periods: Pre-implementation = occurring before implementation date (31 August 2020); Spanning implementation = periods that include the implementation date; Post-implementation = entire period occurred after implementation date

Of the 43,054 clients attending the services at least once before and once after the start of implementation, there were 6240 clients with two or more AUDIT-C screens per year (a total of 20,969 AUDIT-C records in 8173 annual periods). Of these, there were 841 clients (2.0% of the 43,054 clients) who had 1050 frequently screened annual periods (4 + screens per period) and these came from 10 of the 22 participating services. There were 5096 AUDIT-C screens within these periods, accounting for 10.2% of all AUDIT-C screens recorded for the 43,054 clients. The early-support arm appeared to have a lower proportion of frequently screened clients than waitlist control (Fig. 2).

#### Were frequently screened clients high-risk as indicated by AUDIT-C or biomarkers?

Among the 841 frequently screened clients, 181 did not have an HbA1c record, 81 did not have a GGT record, and one did not have a BP record. All clients had at least one of these three biomarkers recorded at least once. More than half (51.6%) had no record of an elevated biomarker or risky AUDIT-C levels, 25.3% had risky drinking, 32.9% had at least one elevated biomarker and 9.9% had both risky drinking and an elevated biomarker (Table 4).

**Table 4:** Frequently screened clients with at least one elevated biomarker by drinking risk (n = 841)

	Non-risk AUDIT-C drinking level (%)	Risky^b^ AUDIT-C drinking level (%)	Total (%)
No record of an elevated biomarker^a^ (%)	51.6	15.5	67.1
Record of 1 + elevated biomarker (%)	23.1	9.9	32.9
Total	74.7	25.3	100.0

^a^Here a biomarker refers to either GGT, BP or HbA1c above the reference range

^b^Risky AUDIT-C drinking level for Aboriginal and Torres Strait Islander populations: AUDIT-C 4 + in males and 3 + in females

There were nearly three times more females than males among the frequently screened group, and 2–3 times more females than males in this group had with a biomarker recorded. Males were older than females (median age: 47 and 32 years respectively). A greater percentage of males had one or more biomarkers above normal levels than females (35%, 22% respectively) and AUDIT-C above the cut-off (49%, 28% respectively) [17–20]. Males also had a higher proportion of individuals with elevated BP or HbA1c than females. However, both sexes had similar proportions of clients with elevated GGT (Table 5).

**Table 5:** Characteristics of frequently screened^a^ clients: gender by elevated AUDIT-C score or biomarkers (n = 841)

	Number of clients	
	Female	Male
n	625	216
Median age (IQR)	32 (28)	47 (22)
With AUDIT-C elevated* (%)	172 (28)	105 (49)
With 1 + elevated biomarker	137 (22)	76 (35)
With systolic BP record	624	216
BP elevated (%)	71 (11)	48 (22)
With HbA1c record	498	162
HbA1c elevated (%)	147 (30)	73 (45)
With GGT record	573	187
GGT elevated (%)	224 (39)	80 (43)

^a^frequently screened = clients with 4 + screens per annual period. Elevated indicates a result above a range that is considered normal: BP (systolic blood pressure) 140 mmHg and above; HbA1c (Haemoglobin A1c) 6.5% and above; GGT (Gamma-Glutamyl Transferase) male: 51 U/L and above, female: 36 U/L and above; AUDIT-C (Alcohol Use Disorders Identification Test—Consumption) for Aboriginal and Torres Strait Islander populations 4 and above in males and 3 and above in females

## Discussion

Previous studies have shown that service-level implementation strategies can improve screening rates in primary care [9]. However, to our knowledge this is the first controlled trial to report on the effect of a service-level alcohol screening implementation program on screening regularity, in addition to screening rate. We previously showed that the odds of a patient being screened in any 2-month period significantly increased during the 24 months of implementation of support in this cluster randomised trial [12]. However, in this report we show that this increase in screening rate was not reflected in an increase in odds of previously unscreened clients in the early-support arm being screened for the first time or of clients receiving regular annual screening when compared to controls. We have also found, in some instances, clients may have received unnecessarily frequent screening. For example, among the 2% of clients who were screened frequently (4 or more times annually), we found that there more women than men, and these women were younger than the frequently screened men. These results together show that service-level strategies successful in increasing overall alcohol screening rates may not lead to screening patterns that are in line with recommendations.

### First-time screening and annual screening

Our sample of 43,054 current clients (i.e., those who attended their services both before and after the implementation of support) accounted for most of the instances of AUDIT-C screening in the broader trial (81.7% or 49,912 instances). However, while over 24 months of implementation there was some increase in the odds of any one client being screened for the first time, this was not significant. There was also no effect on the odds of receiving regular annual screening. These results are explained by the distribution of screening records among the clients. Nearly half of these regular clients (20,141) had no AUDIT-C record at all. In contrast 14.5% (6240) had two or more screens in at least one annual period (accounting for 42% of the screens among the 43,054 clients). Mean AUDIT-C scores in screened individuals at baseline in both study arms of the overall trial (Additional file 1: Table S1) suggest that screening was not specifically targeting people known to drink heavily (early-support = 3.6, waitlist controls 3.3). This is in keeping with recommendations for universal screening. Repeated screening therefore was likely opportunistic—when the clients presented at the service for any reason or as part of scheduled health checks [27].

A range of factors may have contributed to the fact that the efforts of the services did not result in increased first-time or annual screening. Strategies and processes adopted by the services to increase screening differed. Some services reported trying to implement alcohol screening before a medical consultation for all clients, for example by an Aboriginal or Torres Strait Islander health worker or nurse. Screening approaches could also differ among individual clinicians. However, whether a client presents and the nature of their presentation influences whether the client is screened or not. For example, if a client presents in a crisis e.g., with severe asthma or bereavement, taking the opportunity to screen for usual alcohol use may be inappropriate. Also, if a client is known to have current severe alcohol dependence, then screening may be deemed unnecessary.

At a systems level, time pressures and high staff turnover prevalent in this health sector [28] may have contributed to the inconsistent alcohol annual and first-time screening observed in this study. While training resources were available on the website (component 7), the support did not itself include periodic retraining or training with new staff members.

Cultural barriers to screening may also play a role, particularly as alcohol can be a sensitive topic [29]. For example, Aboriginal and Torres Strait Islander peoples may be more comfortable to discuss private or sensitive issues with people of the same gender [30]. So, a male patient may be less comfortable talking with a female clinician. Further, cultural compatibility between clinician and client can be important when making clinical assessments relating to mental health of Aboriginal Australians [30]. So, some clients may be less willing to accept questions about alcohol from clinicians who are not Aboriginal or Torres Strait Islander, particularly if they do not know them well. On the other hand, community controlled services operate in closely knit communities so there may be instances in which family relationships or cultural restrictions impact on Aboriginal and Torres Strait Islander health workers undertaking screening with some clients, contacts or relatives [31] for drinking.

### Was frequent screening appropriate?

National guidelines for preventative care for Aboriginal and Torres Strait Islander peoples recommend more frequent alcohol screening of high-risk groups [10]. However, there is no agreed definition of the appropriate frequency of screening. Aboriginal and Torres Strait Islander peoples experience higher rates of chronic diseases than the general Australian population [32]. Monitoring of alcohol consumption may be needed for clients with health conditions that can be exacerbated by alcohol, such as viral hepatitis. These conditions may also require more frequent visits, which present an opportunity for more frequent use of an alcohol screening tool. The 841 clients (2% of current clients) who were screened four or more times in at least one annual period accounted for nearly 11% of screens recorded for current clients. However, in most cases our data did not reveal why those clients were more frequently screened—less than half had elevated biomarkers or AUDIT-C levels.

Females were overrepresented in the frequently screened sample, even though a smaller percentage of females had elevated biomarkers and elevated AUDIT-C than males in this group. They also had a much lower median age. This could suggest that frequent screening was occurring in healthy females, possibly during regular antenatal or reproductive health consultations (e.g., for contraception). Other reasons for frequent health service contact in healthy women could be visiting with dependents, or a greater willingness to seek preventive care or routine health checks than males. Conversely, the higher proportions of individuals with elevated biomarkers in the smaller sample of frequently-screened males might reflect this group seeking healthcare for treatment or monitoring of health conditions more often than attending for a preventive health check.

### Recommendations for policy, practice, and research

Annual screening allows the clinician to detect unhealthy drinking earlier and offer treatment or support, including brief intervention [10, 33]. Individuals’ drinking patterns can vary over time [34, 35], including over relatively short periods [36]. Further, intermittent drinking patterns can be common among Aboriginal and Torres Strait Islander peoples [7], with drinking triggered by events such as ‘Sorry Business’ (grieving after a bereavement). This means that annual screening rather than one-off is a better way to gain a picture of the individual’s drinking.

In Australia, primary care services for Aboriginal and Torres Strait Islander peoples report on rates of risky drinking based on AUDIT-C scores as part of their national key performance indicators [17]. AUDIT-C is particularly suitable in this setting as it is brief and has been validated in primary care [11]. It has also been shown to be acceptable to Aboriginal and Torres Strait Islander peoples, though its delivery may need to be adapted for local culture and context [37]. However, regular screening with AUDIT-C or any other validated instrument is not otherwise mandated or widely promoted. For example, screening with a validated instrument such as AUDIT-C is not included in the Royal Australian College of General Practitioners’ annual health check templates. Similarly, such screening is not required for the Medical Benefits Scheme to reimburse services for the conduct of the annual health assessment for Aboriginal and Torres Strait Islander peoples [27]. The health assessment uptake rates vary from about 20% per year in 15–24 year olds to over 40% in people aged 65 and older [38]. These rates are greater than the AUDIT-C screening rates shown in this study (12.7% based on 18-months’ baseline, Additional file 1: Table S1). So, incorporating AUDIT-C in this assessment could improve detection and increase annual screening rates.

Refining strategies to reach unscreened adults should be considered. In addition to screening when clients present to the service, opportunities for outreach alcohol screening could be employed. For example, some ACCHS already engage their clients through activities outside of the clinic setting, which takes various forms, including camps or community gatherings [39]. Indeed, some participating services mentioned conducting screening in the community and updating records in practice software. Strategies to support these kinds of activities should be included in future designs.

Results of this analysis show that successful strategies designed to increase universal alcohol screening may lead to repeated screening of a particular group of clients rather than necessarily increasing screening in an unscreened or underscreened group. As alcohol use is a sensitive topic [29], broaching the subject too frequently could lead to the client becoming irritated or avoiding contact with the service. So, it is important that services and research studies clarify an optimal frequency of screening or monitoring for different clinical situations. Initiatives designed to improve screening and treatment for unhealthy alcohol use should ensure that training and monitoring of outcomes include consideration of the appropriate frequency and regularity of screening.

Identifying and addressing potential clinician and client barriers to screening, including those determined by cultural norms should be incorporated into improvement programs. Programs that incorporate audit-and-feedback cycles such as those based on continuous quality improvement (CQI), would be suited to this due to their iterative nature. After the initial cycle that aims to increase implementation of screening, these programs could then focus on screening regularity. Practice software can be an important tool in these efforts. Aside from its role in providing data for monitoring service improvement, services could choose to implement further modifications to help facilitate more appropriate screening frequency such as automated screening reminders based on clients’ prior records [40–42]. However, services participating in this study have pointed out that too many reminders can interfere with clinical practice.

### Limitations

This study is a post-hoc analysis of data from a larger trial [13], to explore potential unwanted effects of the intervention, and to scrutinize if the benefits were as significant as they appeared [13]. More than half of that clients from that broader study population were excluded from the analysis because they had not attended the service during the baseline period. A longer baseline could have allowed more clients to be included in analyses investigating first-time screening. The same limited availability of pre-implementation data prevented us from determining the levels of annual screening before implementation and therefore we had to limit this analysis to the comparison of study arms post-implementation. However, our assumption of the absence of regular annual AUDIT-C screening is supported by generally low screening levels at baseline.

While the training provided to services emphasised annual screening of clients as best-practice, several aspects of the support model may have resulted in efforts that prioritised increasing number of screening events rather than increasing annual or first-time screening. These included suggestions for opportunistic screening, including during pre-consultation examinations or antenatal checks. Further, regular feedback reports presented a graph of change in bi-monthly screening rates (as well as summary of screening in the last 12-months). They did not include details on first-time screening events, or over-frequent screening of clients. The duration of the trial would not have allowed for reporting on effect of the intervention on numbers of clients screened annually. Barriers and facilitators of appropriate screening regularity were not specifically discussed during champion teleconferences.

### Conclusions

Despite previously demonstrating a marked increase in the odds of screening occurring in any 2-month period with service-wide support, we were unable to show significant increases in clients screened for the first time or in annual screening of clients over 24 months of implementation of support. This appeared to be due to an uneven distribution of screening, with a small percentage of clients being screened more frequently than annually while nearly half clients were not screened at all. Females tended to be overrepresented among the more frequently screened clients. Further strategies to improve alcohol screening should focus on appropriate regularity as well as rate of screening in order to garner clinically useful information about alcohol consumption.

## Supplementary Information

**Additional file 1: Figure S1.** Construction of client’s annual periods for annual screening analysis (question 2). **Figure S2.** Construction of client’s annual periods for a client screened four or more times annually (question 3). **Figure S3.** Unadjusted first‐time screening rates for the 22 services over 24 months of implementation, by study arm and by service. **Figure S4.** Unadjusted annual screening rates for the 22 services over 24 months of implementation, by study arm and by service. **Table S1.** Full trial sample at baseline: characteristics by trial arm. **Table S2.** Fixed effects of the support model on the odds of screening in previously unscreened clients **A** without control variables; **B** with control variables of age and gender. **Table S3.** Fixed effects of the support model on the odds of receiving annual screening **A** without control variables; **B** with control variables of age and gender.

## Data Availability

The datasets generated and/or analysed during the current study are not publicly available due to ethics restrictions. Data can only be made available upon additional approvals from the ethics committees and consent from the 22 participating services.

## Abbreviations

ACCHS: Aboriginal Community Controlled Health Services (ACCHS)
AUDIT-C: Alcohol Use Disorders Identification Test—consumption
BP: Systolic blood pressure
CI: Confidence interval
GGT: Gamma-glutamyltransferase
HbA1C: Haemoglobin A1c
ICC: Adjusted intraclass correlation coefficient
