# Supplementary material for: Effects of service-wide support on regularity of alcohol screening of clients in Australian Aboriginal and Torres Strait Islander Community Controlled Health Services: a cluster randomised trial
Source: Addict Sci Clin Pract. 2022 Feb 19;17:13. doi: 10.1186/s13722-022-00294-6 (PMC8858520; doi:10.1186/s13722-022-00294-6)
Supplement: Supplementary file 1 — Additional file 1: Figure S1. Construction of client’s annual periods for annual screening analysis (question 2). Figure S2. Construction of client’s annual periods for a client screened four or more times annually (question 3). Figure S3. Unadjusted first‐time screening rates for the 22 services over 24 months of implementation, by study arm and by service. Figure S4. Unadjusted annual screening rates for the 22 services over 24 months of implementation, by study arm and by service. Table S1. Full trial sample at baseline: characteristics by trial arm. Table S2. Fixed effects of the support model on the odds of screening in previously unscreened clients A without control variables; B with control variables of age and gender. Table S3. Fixed effects of the support model on the odds of receiving annual screening A without control variables; B with control variables of age and gender. [file 13722_2022_294_MOESM1_ESM.pdf]

EFFECTS OF SERVICE-WIDE SUPPORT ON REGULAR ALCOHOL SCREENING OF CLIENTS IN AUSTRALIAN ABORIGINAL AND TORRES STRAIT ISLANDER COMMUNITY CONTROLLED HEALTH SERVICES – A CLUSTER RANDOMISED TRIAL

ADDITIONAL MATERIAL

Monika Dzidowska[1,2], Jacques E Raubenheimer[3], Timothy A Dobbins[4] , KS Kylie Lee[1,2,5,6] , Noel Hayman[7,8,9] , Julia Vnuk[10,11], Paul Haber[1,2], Katherine M Conigrave[1,2]

[1]The University of Sydney, Faculty of Medicine and Health, Discipline of Addiction Medicine, NHMRC Centre of Research Excellence in Indigenous Health and Alcohol, Sydney, Australia. ADDRESS: Lev 6, King George V Building (C39), The University of Sydney, NSW 2006
[2]The Edith Collins Centre (Translational Research in Alcohol Drugs and Toxicology), Sydney Local Health District, Australia. ADDRESS: Drug Health Services, Royal Prince Alfred Hospital (KGV), 83-117 Missenden Road, Camperdown, NSW 2050
[3]The University of Sydney, Faculty of Medicine and Health, Translational Australian Clinical Toxicology Program, Sydney, Australia. ADDRESS: Lev3, 1-3 Ross Street (K06), The University of Sydney, NSW 2006
[4]University of New South Wales, School of Public Health and Community Medicine, Sydney, Australia. ADDRESS: Level 3, Samuels Building Gate 11, Botany Street, UNSW, NSW 2052
[5]National Drug Research Institute, Faculty of Health Sciences, Curtin University, Perth, Australia, ADDRESS: 7 Parker Place, Bentley WA 6102
[6]La Trobe University, Centre for Alcohol Policy Research, Melbourne, Australia. ADDRESS: NR1, La Trobe University, Bundoora, VIC 3086
[7]Southern Queensland Centre of Excellence in Aboriginal and Torres Strait Islander Primary Health Care (Inala Indigenous Health Service), Brisbane, Australia. ADDRESS: 37 Wirraway Parade, Inala QLD 4077
[8]Griffith University, School of Medicine, Gold Coast, Australia. ADDRESS: Griffith Health Centre (G40), Gold Coast campus, Griffith University QLD 4222
[9]University of Queensland, School of Medicine, Brisbane, Australia. ADDRESS: Herston Road, Herston QLD 4006
[10]Aboriginal Health Council of South Australia, Adelaide, Australia. ADDRESS: 220 Franklin Street, Adelaide SA 5000
[11]Adelaide Rural Clinical School, The University of Adelaide, Adelaide, Australia. ADDRESS: Level 1, Helen Mayo North Frome Road, Adelaide SA 5005

Figure S1. Construction of client's annual periods for annual screening analysis (question 2)

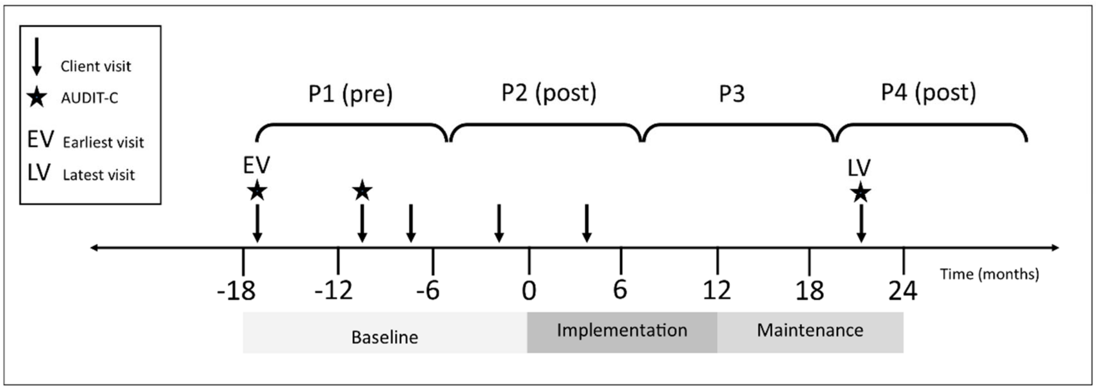

This figure illustrates construction and classification of four annual periods for a client with six service visits, three of which included AUDIT-C screening (designated with a star). Annual periods (P1 – P4) are constructed based on the earliest available client visit date (EV). A period is classified as occurring pre or post implementation based on the latest visit date within the period. Periods occurring pre-implementation are not used in the analysis. The client is classified as annually screened if all post-implementation periods with visits contain at least one AUDIT-C screen. Since this client presented to the service at two post periods (P2, P4) but only one contains AUDIT-C screening, they are classified as not annually screened.

Figure S2. Construction of client's annual periods for a client screened four or more times annually (question 3)

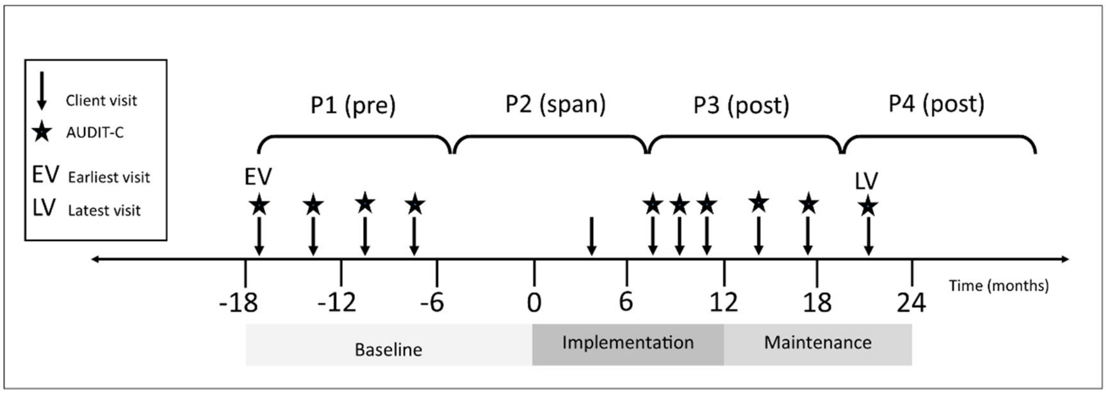

This figure illustrates construction and classification of four annual periods for a client with 11 service visits, 10 of which included AUDIT-C screening (designated with a star). Annual periods (P1 – P4) are constructed based on the earliest available client visit date (EV). A period is classified as occurring pre, span or post implementation based on the commencement and end dates of that period (i.e., the anniversary of EV). The client is classified as frequently screened if at least one period contains four or more visits with AUDIT-C screening. Since P1 and P3 contain four or more visits with AUDIT-C, this client is classified as frequently screened.

Figure S3. Unadjusted first-time screening rates for the 22 services over 24 months of implementation, by study arm and by service.

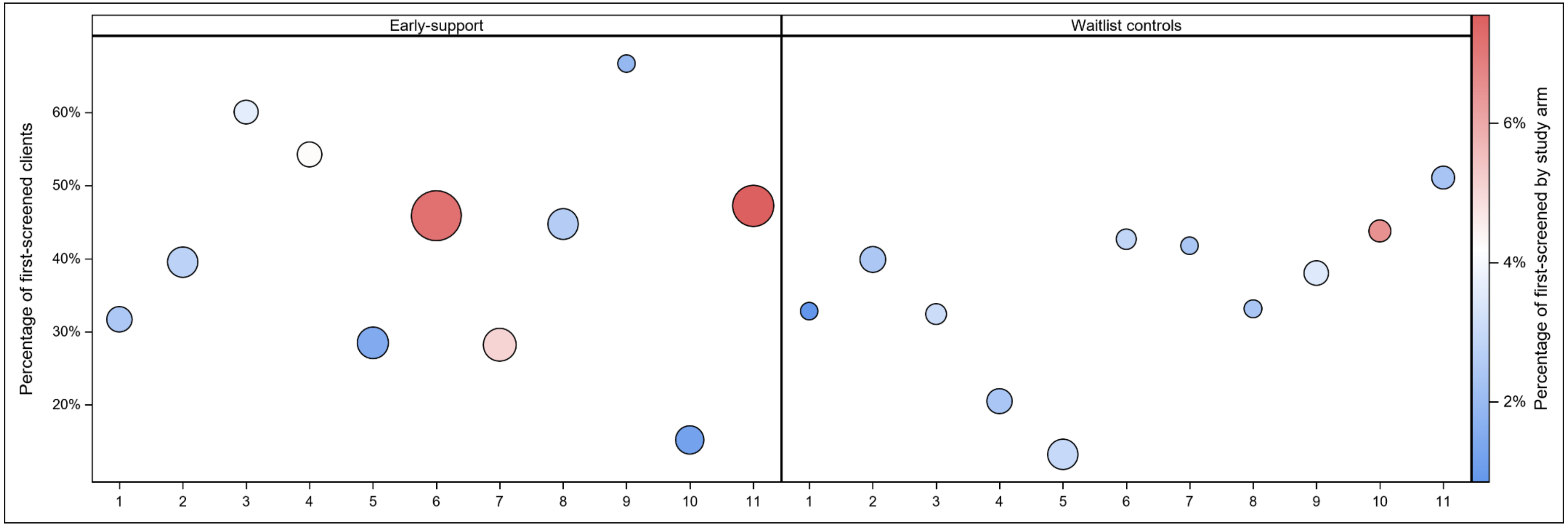

Services are represented by numbers 1-11 in each study arm (x-axis).
The size of the bubble represents the overall size of the service, based on the number of clients in the overall trial sample.
The vertical position of the bubble represents the percentage of first-screened clients in each service (denominator is the number of clients from that service that were included in sample for assessing first-screening). The corresponding y-axis is on the left.
The colour of the bubble represents the percentage of first-screened clients in that the service's study arm that were contributed by that service (denominator is the number of clients in that study arm that were included in the sample to assess first-screening). The corresponding y-axis is on the right.

Figure S4. Unadjusted annual screening rates for the 22 services over 24 months of implementation, by study arm and by service.

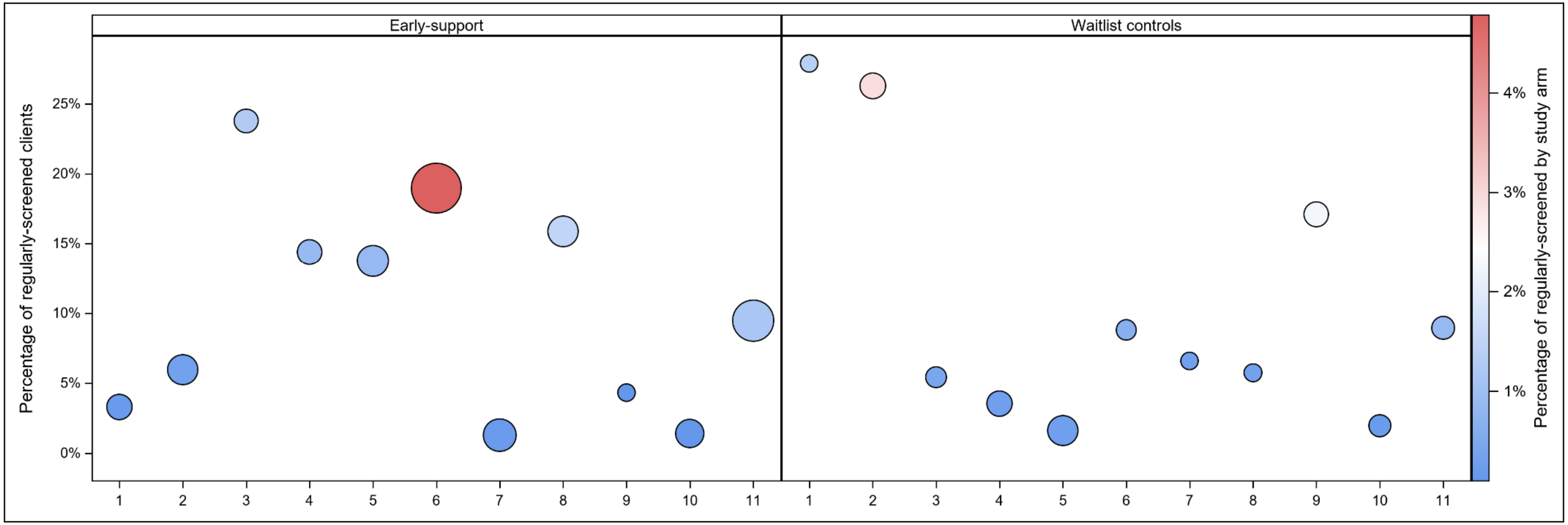

Services are represented by numbers 1-11 in each study arm (x-axis).
The size of the bubble represents the overall size of the services, based on the number of clients in the overall trial sample.
The vertical position of the bubble represents the percentage of annually screened clients in each service (denominator is the number of clients from that service that were included in the sample for assessing annual screening). The corresponding y-axis is on the left.
The colour of the bubble represents the percentage of annually screened clients in that the service's study arm that were contributed by that service (denominator is the number of clients in that study arm that were included in the sample to assess annual screening). The corresponding y-axis is on the right.

Table S1. Full trial sample at baseline: characteristics by trial arm

Characteristic	Early support	Waitlist control
**Services**		
N	11	11
Mean clients per service (SD)	3678 (2380)	1967 (791)
Remoteness		
Urban and inner regional	5	5
Outer regional and remote	2	3
Very remote	4	3
**Clients**		
N	40,459	21,631
Mean age of clients in years (SD)	37.1 (16)	37.4 (16.3)
Number of female clients (%)	22342 (55.2)	11938 (55.2)
Mean observations[b] per client (SD)	3.5 (2.6)	3.5 (2.6)
Clients screened with AUDIT-C (%)	7213 (17.8)	4601 (21.3)
Mean AUDIT-C score[c] (SD)	3.6 (3.6)	3.3 (3.4)
Clients with an AUDIT-C>0[c] (%)	4092 (56.7)	2811 (61.1)

[a]Baseline period: from 28.02.2016 to 30.08.2017 inclusive. [b]An observation appeared in the dataset for a client if they attended their service for a consultation in the preceding two-month reference period at least once. [c]The denominator is the number of clients who had at least one recorded AUDIT-C score.

Table S2. Fixed effects of the support model on the odds of screening in previously unscreened clients (A) without control variables; (B) with control variables of age and gender

A	Effect	Estimate [95% CI]	p
	Odds of outcome in waitlist controls	0.52 [0.37-0.74]	<0.001
	Odds Ratio: Condition (Early-support)	1.33 [0.81-2.18]	0.25
	Intracluster correlation coefficient (ICC)	0.1	

B	Effect	Estimate [95% CI]	p
	Odds of outcome in waitlist controls	0.51 [0.36-0.73]	<0.001
	Odds Ratio: Condition (Early-support)	1.33 [0.81-2.19]	0.26
	Odds Ratio: Gender (male)	0.96 [0.92-1.01]	0.13
	Odds Ratio: Age	1.00 [1.00-1.00]	0.2
	Intracluster correlation coefficient (ICC)	0.1	

Table S3. Fixed effects of the support model on the odds of receiving annual screening (A) without control variables; (B) with control variables of age and gender

A

Effect	Estimate [95% CI]	p
Odds of outcome in waitlist controls	0.08 [0.04-0.15]	<0.001
Odds Ratio: Condition (Early-support)	0.99 [0.42-2.37]	0.98
Intracluster correlation coefficient (ICC)	0.24	

B

Effect	Estimate [95% CI]	p
Odds of outcome in waitlist controls	0.08 [0.04-0.16]	<0.001
Odds Ratio: Condition (Early-support)	0.99 [0.42-2.33]	0.98
Odds Ratio: Gender (male)	1.12 [1.05-1.20]	<0.001
Odds Ratio: Age	1.00 [0.99-1.00]	<0.01
Intracluster correlation coefficient (ICC)	0.24	
